# The Quantitative Profiling of Oxylipins from Arachidonic Acid by LC-MS/MS in Feces at Birth 3 Days and 21 Days of Piglets

**DOI:** 10.3390/metabo12080702

**Published:** 2022-07-28

**Authors:** Ningning Huang, Xiangchen Liu, Xiaoqi Pei, Jian Peng, Hongkui Wei

**Affiliations:** 1 Department of Animal Nutrition and Feed Science, College of Animal Science and Technology, Huazhong Agricultural University, Wuhan 430070, China; huangning2020@webmail.hzau.edu.cn (N.H.); liuxc_88@webmail.hzau.edu.cn (X.L.); peixq@webmail.hzau.edu.cn (X.P.); pengjian@mail.hzau.edu.cn (J.P.); 2 The Cooperative Innovation Center for Sustainable Pig Production, Wuhan 430070, China

**Keywords:** arachidonic acid, oxylipins, LC-MS/MS, 12-HETE, chiral analysis, suckling piglets

## Abstract

Oxylipins (also called eicosanoids) are enzymatically or nonenzymatically generated by oxidation of arachidonic acid (ARA) and are major mediators of ARA effects in the body. Previous studies demonstrated the importance of ARA in infant growth, brain development, immune response, and health. With the developments in lipidomic methodologies, it is important for exploring more ARA-deprived oxylipins to better understand the physiological functions of ARA. The concentrations of oxylipins in feces were determined from days 3 to 21 postnatally of suckling piglets in vivo. Feces were collected at two critical time points of the suckling piglets (3d and 21d after birth) and about 48 oxylipins were analyzed by using a target metabolomics approach based on Liquid Chromatography-Tandem Mass Spectrometry (LC-MS/MS). Here, 21 oxylipins were derived from ARA, and 11 differential oxylipins (Log2|fold change| ≥ 1.0) at birth 3d and 21d were identified. Particularly, 12-HETE was more abundant in feces at birth 3 days rather than 21 days. Considering that 12-HETE was a racemic mixture of stereoisomers containing the *S* and *R* enantiomers, we further detected the concentrations of 12(S)-HETE and 12(R)-HETE between the two time points by chiral LC-MS/MS analysis. There was no significant difference in the concentrations of 12(S)-HETE and 12(R)-HETE. It was showed that ARA - derived oxylipins might be related to the physiological changes of piglets during growing. Our results provided new information for describing the physiological changes of the piglets over the suckling period.

## 1. Introduction

Oxylipins are mainly the oxidation products of n-3 and n-6 long chain polyunsaturated fatty acids (LCPUFA) and found throughout the body in all tissues, urine, and blood. It is well known that oxylipins are eicosanoids produced from the n-6 PUFA, arachidonic acid (ARA) (20:4n-6). ARA is normally present in membrane phospholipids. The exposure to different physiological and pathological stimuli triggered the release of ARA from cell phospholipids through the activity of phospholipase A2 (PLA2). Free ARA can be catalyzed to form oxylipins via cyclooxygenase (COX), lipoxygenase (LOX), and cytochrome P450 (CYP450) enzymes pathways [1,2]. COX enzymes are comprised of two different isomers of COX, namely, COX-1 and COX-2, and have dioxygenase activity. COX enzymes can provide two oxygen molecules to the fatty acid substrate to produce a 5-carbon ring structure at the 8 to 12 carbon positions of 20-carbon fatty acids, producing prostanoids (PGs) and thromboxanes (TXs) [3]. Similarly, LOX can also donate two coxygen molecules to ARA, resulting in the formation of hydroxy derivatives, including leukotrienes (LTs), hydroperoxyeicosatetraenoic acids (HPETEs), hydroxyeicosatetraenoic acids (HETEs), and lipoxins (LXs). CYP enzymes metabolized ARA to produce HETEs, epoxy-eicosatrienoic acids (EETs), and dihydroxy-eicosatetraenoic acids (DHETs) [4].

Oxylipins were not only formed by these three major enzymatic pathways, but also by non-enzymatic oxidation autoxidation. They can be formed by microsomal prostaglandin E synthase (mPGES) or soluble epoxide hydrolase (sEH) in ARA cascade [2]. Isoprostanes (IsoPs), lipid peroxidation products produced through the free radical-mediated oxidation of ARA are usually used as biomarkers of oxidative stress in vivo [5]. Moreover, it has been recognized that HETEs are generated by peroxidase (POX) activity [6].

It was reported that ARA is critical for infant growth, immune response and health, and it has been added to infant formulas for more than two decades [7]. Importantly, these functions of ARA are mainly mediated by oxylipins. Additionally, a growing number of studies have confirmed that oxylipins are involved in regulating intestinal innate immunity [8], inflammation [9], injury and disease [10]. Oxylipins are media to understand the effects of ARA as they act as metabolites of ARA. With the developments in lipidomic methodologies, it is important for exploring more ARA-deprived oxylipins.

The suckling period is a critical part of piglets that is characterized by rapid morphological and physiological modifications, which include the development of the intestine and immune system, significant muscle protein deposition, and the beginning of fat deposition, subsequently, affecting the performances of the weaned animals [11]. A previous study described that the dietary intake of ARA affected plasma oxylipins levels in suckling piglets [12]. Oxylipins have enormous heterogeneity associated with a number of oxidation pathways, and the molecules structure of many oxylipins is similar. It is important to quantify the profile of oxylipins. Studies on oxylipins have primarily concentrated on milk or serum of mice and humans in health or disease, but the level of oxylipins has not previously been explored in piglets, especially growing piglets.

In this study, we monitored the comprehensive lipid profiles of feces in piglets by applying a targeted metabolomic approach based on mass spectrometric. We had detected about 48 oxylipins to disclose the metabolic changes that could describe the biological developments of suckling pigs in two different stages. Of the 48 oxylipins, 21 oxylipins derived from ARA and quantitative analysis of the difference multiple changes of oxylipins showed that some oxylipins from ARA were different in feces in two different stages. Specifically, 12-HETE was the highest content of ARA-derived oxylipins at 3 days after birth, and we further indicated that there was no significant difference between *S* and *R* enantiomers in feces of suckling piglets.

## 2. Results

### 2.1. Oxylipins Profile in Feces of Suckling Piglets

**Figure 1:**
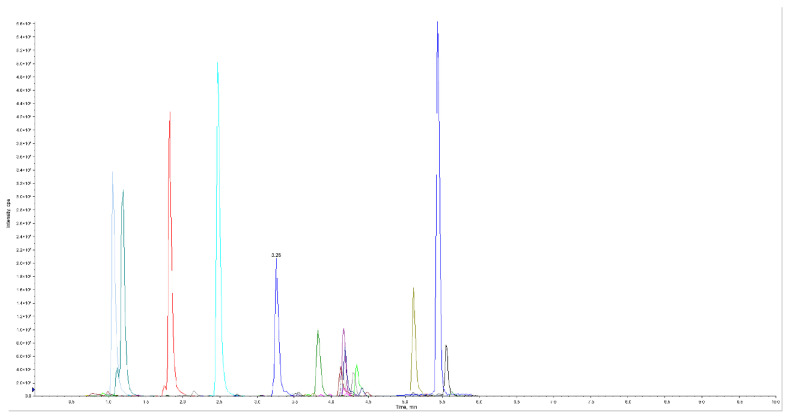
LC-MS/MS chromatogram of 48 oxylipins performed on a triple quadrupole employing dynamic MRM (*n* = 10). MRM metabolite detection multipeak diagram showed the substances that could be detected in the sample, with the mass spectrum peak of each color representing one metabolite detected.

**Figure 2:**
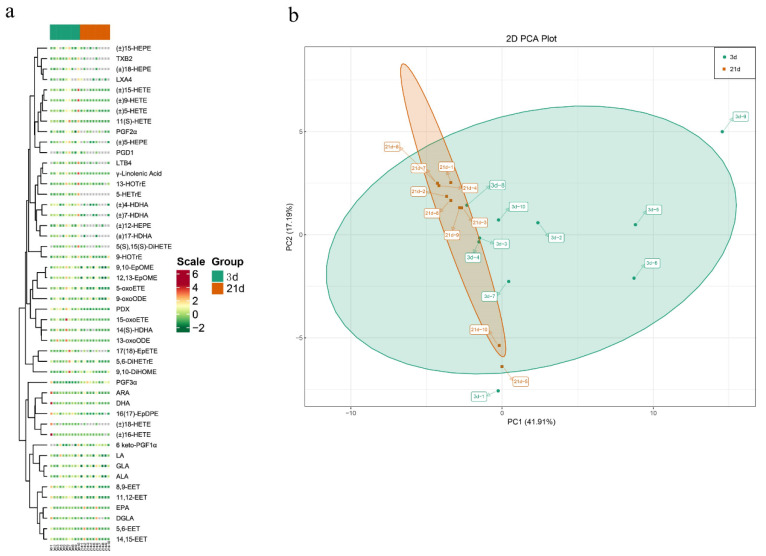
Oxylipins were detected in feces of suckling piglets at birth day 3 and day 21 (*n* = 10). (**a**) Heat map hierarchical clustering of detected all metabolites in this study. Hierarchical trees were drawn based on detected metabolites in feces of suckling piglets at birth day 3 and day 21. The columns correspond to feces at 3d and 21d postnatally, while the rows represent different metabolites. (**b**) Principal component analysis for metabolites identified in feces.

Previous studies have reported that LC-MS/MS method has high sensitivity and is used to analyze lipid or their mediators, and allows the identification of oxylipins with structural similarity [13]. In the present study, we profiled the changes of lipidomics in feces of piglets at 3 and 21 days after birth. Our LC-MS/MS method, employing multiple reaction monitoring (MRM), evaluated oxylipins in feces (Figure 1). We were able to detect 48 oxylipins through the targeted quantitation method in feces (Figure 2a). These oxylipins were grouped into eight major classes, including ARA, eicosapentaenoic acid (EPA), docosahexaenoic acid (DHA), Linoleic Acid (LA), α-Linolenic Acid (ALA), Dihomo-γ-Linolenic Acid (DGLA), γ-Linolenic Acid (LA), and mead acid (MA). Overall, the number of oxylipins derived from ARA was the largest and contained 21 oxylipins, followed by EPA and DHA, which had seven oxylipins, respectively. Very few metabolites belonged to the classes of Dihomo-γ-Linolenic Acid (DGLA), γ-Linolenic Acid (LA), and mead acid (MA) (Table 1). Moreover, we observed that metabolic data from the early postnatal period (3d) was clearly separated from those from before weaning (21d) (Figure 2a). Based on the PCA, a clear separation between the two different time points could be observed by the PC1. Furthermore, the PC2 distinctly distinguished the feces of piglets at 3 and 21 days after birth. These results, depicting 59.10 variations with PC1 (41.91%) and PC2 (17.19%), also verified the reliability of the metabolome dataset (Figure 2b).

### 2.2. Differential Oxylipins in Feces of Suckling Piglets

**Figure 3:**
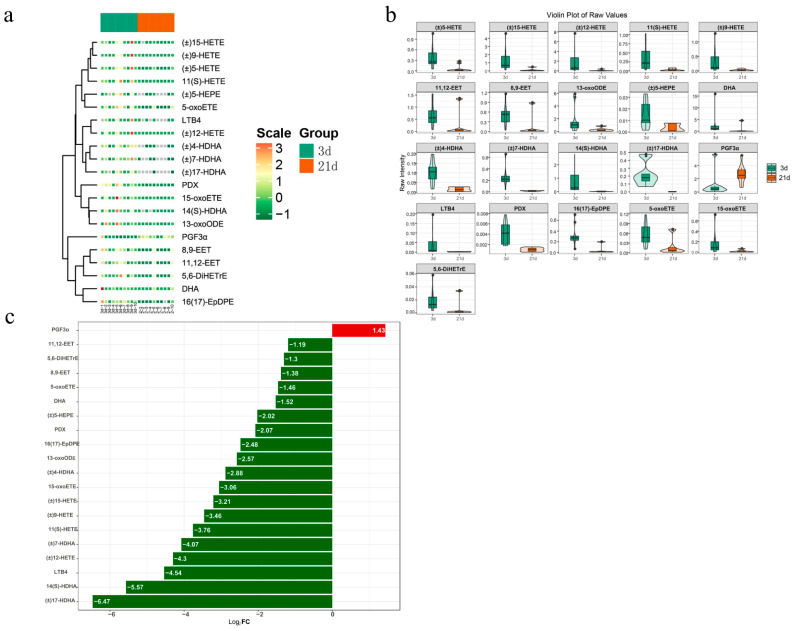
Differential oxylipins were analyzed in feces between 3d and 21d postnatally (*n* = 10). (**a**) Heatmap analysis of detected 21oxylipins in this study. (**b**) Violin plot analysis of 21 differential 21oxylipins in this study. The *x* axis indicates the name of the groups, and the *y* axis indicates the expression quantity. (**c**) The bar plot of top 20 up-regulated and down-regulated oxylipins.

In order to understand the changes of oxylipins in feces of piglets at different developmental stages, we compared oxylipins in feces at 3 and 21 days of age. Our profiling experiments revealed 21 differential oxylipins (Log2|fold change| ≥ 1.0) (Figure 3a). Of these 21 oxylipins, 20 oxylipins were significantly downregulated at 21 days of age comparing to 3 days of age. However, only one oxylipin was upregulated (Figure 3b). Furthermore, quantitative analysis of the difference multiple changes of oxylipins showed that the highest degree of down-regulation (Log2FC = −6.47) was (±)17-HDHA, followed by 14(S)-HDHA, LTB_4_ and (±)12-HETE (Figure 3c). This suggested that some oxylipins were more abundant during the early life of piglets, and decreased with age.

### 2.3. Arachidonic Acid-Derived Oxylipins in Feces

**Figure 4:**
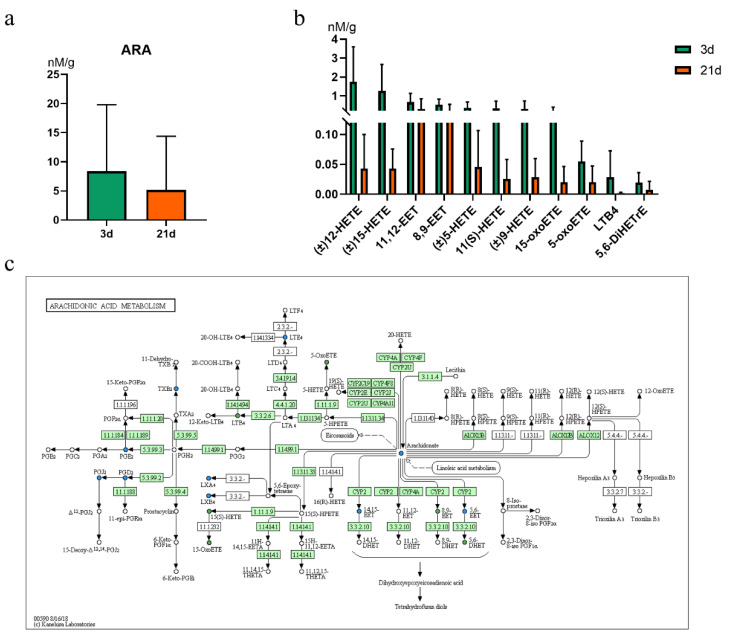
The quantitative content of ARA derived differential oxylipins in feces (*n* = 10). (**a**) the quantitative content of ARA in feces of suckling piglets at birth day 3 and day 21. (**b**) the absolute levels of ARA derived differential oxylipins. (**c**) KEGG pathway analysis of the ARA derived differential oxylipins in feces at 3 and 21 days after birth. Blue indicates that substances were detected but had no significantly change compared to these of 21d, and green indicates that the content of oxylipins was significantly down-regulated in feces from 21 days after birth.

These differential oxylipins were further classified and presented in Table 2. Most of the differential oxylipins were derived from ARA, and we then focused on ARA-derived oxylipins. We further compared changes in ARA and its oxylipins in feces by statistical analysis. There was a change in concentration of ARA in feces of suckling piglets through comparing two developmental stages (at birth 3d and 21d) (Figure 4a). The quantitative contents of 11 ARA-derived oxylipins in feces were shown in Figure 4b. Overall, these oxylipins from ARA were higher in feces of 3d compared with that of 21d. Moreover, of these 11 oxylipins, 12-HETE was the highest level of ARA-derived oxylipins in feces at early age. The KEGG pathway analysis of ARA metabolism further provided a possible reason for the changes of oxylipins at 3 and 21 days after birth (Figure 4c). The KEGG analysis indicated that oxylipins from ARA were primarily produced by LOX and CYP450 pathway. 

### 2.4. Chiral Analysis the S and R Enantiomers of 12-HETE in Feces 

**Figure 5:**
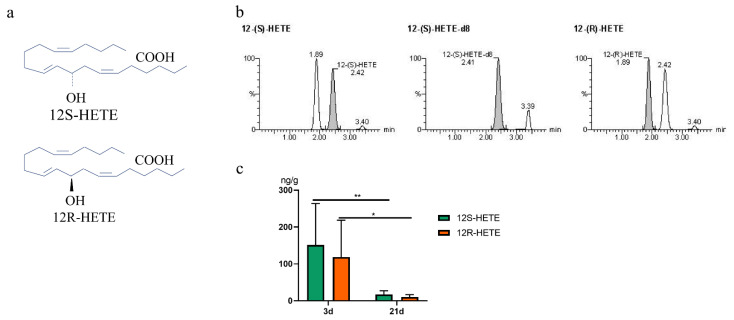
Chiral LC-MS/MS analysis of 12-HETE in feces of suckling piglets (*n* = 10). (**a**) Chemical structure diagram of 12(S)-HETE and 12(R)-HETE. (**b**) Selected reaction monitoring (SRM) chromatograms demonstrating the resolution of 12(S)- and 12(R)-HETE in feces of suckling piglets at birth day 3 and day 21 (*n* = 9). (**c**) the quantitative content of 12(S)- and 12(R)-HETE in feces at 3d and 21d postnatally, values are the mean ± SD, *n* = 10. Statistical analysis was performed by Tukey tests, * *p* < 0.05, ** *p* < 0.01.

12-LOXs, which are enantiomer enzymes, can convert ARA to 12-HETE, resulting in a racemic mixture of stereoisomers containing the *S* and *R* enantiomers (Figure 5a). In addition, CYP450s can also generate12-HETEs, and it was reported that human CYP450s synthesize predominantly *R*-HETEs in vitro [14]. We further detected the concentration of 12(S)-HETE and 12(R)-HETE in feces by chiral LC-MS/MS. The chiral LC-MS/MS method achieved excellent resolution of the enantiomers of *S* and *R* enantiomers (Figure 5b). The *S* enantiomer eluted at 2.42 min, while the *R* enantiomer eluted at 1.89 min, and 12(S)-HETE-d8 eluted at 2.41 min. The absolute levels of 12(S)-HETE and 12(R)-HETE in feces of suckling piglets were showed in Figure 5c and Table 3. There was no significant difference in 12(S)-HETE and 12(R)-HETE concentration at the same developmental stage; however, the level of 12(S)-HETE or 12(R)-HETE in feces of 3d was higher than that of before weaning. The results indicated that 12(S)-HETE or 12(R)-HETE generally presented at the higher levels in the feces at early stage of sucking piglets, and the contribution of the LOX pathway was equivalent to CYP450.

## 3. Discussion

The objective of our study was to investigate the profiling of oxylipins from ARA in the feces of suckling piglets. To our knowledge, this is the first study to assess on the concentrations of ARA derived oxylipins and the *S* and *R* enantiomers in feces of suckling piglets. The use of quantitative metabolomics technology in this study allowed us to characterize the detectable suckling piglets oxylipins metabolome even though the structure of these oxylipins was similarly. The results showed that the oxylipins in feces of piglets varied at different developmental stages, and quantitative analysis of the difference multiple changes of oxylipins showed that some oxylipins from ARA were different in feces during early postnatal development compared with pre-weaning piglets. 

Our study provided foundational data concerning the change of oxylipins profile in feces during the suckling period of piglets by quantitative LC-MS/MS. A total of 48 oxylipins were quantifiable in this study, and nearly half of the oxylipins were derived from ARA, which was similarly with the oxylipins feature of previous studies [15,16]. In addition, we found that some ARA- derived oxylipins generally present at the higher levels at day 3 after birth. Colostrum and milk are the earliest nutrient sources for the newborn piglets, and ARA is the most abundant n-6 PUFA in milk [17]. Mean values of ARA reported in sow milk are 0.7% of total fatty acids [18]. It was absorbed into the intestine after intaking ARA, and the remaining ARA is excreted by the body with feces. Moreover, the intestinal function of newborn piglets is not completely developed, which may contribute to the higher abundance of some oxylipins from ARA at early of life. However, there was no significant difference in the levels of ARA in our study. Noteworthily, large inter-individual differences may be the reason for it. In addition, free ARA can be catalyzed to form oxylipins via three major enzymatic pathways including COX, LOX, and CYP450 and non-enzymatic autoxidation. The ways produced oxylipins may account for 11 differential oxylipins from ARA at two time points, although there were no changes in ARA concentration.

It is a major finding that 12-HETE was the most abundant oxylipins formed ARA at 3 days after birth via targeted analysis in our study. Similarly, young rat lens had the capacity to synthesize12-HETE from exogenous ARA, and 12-HETE synthetic capacity in the 4 day old rat lens was more than that in the 15 day old rat lens [19]. 12-HETE is the LOX- and CYP450-derived ARA metabolite; however, the expression of metabolic enzymes in tissues of newborn animals has not been confirmed.

12-HETE is a racemic mixture of 12(S)- and 12(R)-enantiomers. Corey was the original pioneering synthesis of 12(S)-HETE in 1978 [20], 12(S)-HETE was produced by the 12(S)-LOX pathway, and 12(R)-HETE was a product of either 12(R)-LOX or CYP450 [21]. Moreover, 12S- and 12R-HETE have different physiological function. It reported that 12S-HETE played a direct role in platelet aggregation, cancer, and diabetes [22]. The cornea had been reported to produce 12R-HETE, and it can inhibit Na^+^-K^+^-ATPase activity. It was indicated that 12R-HETE may also have a role in the eyes [23]. Considering that their structures are similar, it is necessary for performing chiral analysis of 12-HETE to detect 12(S)- and 12(R)-HETE concentrations in feces. *S*- and *R*-enantiomers were well resolved in our study. There was no significant difference in 12(S)-HETE and 12(R)-HETE concentrations at the same developmental stage. This suggested that the relative contribution of CYP450 (which produces appreciable *R*-enantiomers) is equivalent to the 12-LOX pathway (which mainly produces *S*-enantiomers). Moreover, it is the first report of the enantiomeric composition of 12-HETE in the feces of suckling piglets in vivo.

There are many factors that could affect the oxylipins profile, including sow milk, feed, microbiota and raising conditions. Piglets were followed from farrowing until 21 days after birth and they were housed under the same condition in order to better analyze the changes of oxylipins in feces of suckling piglets at different developmental stages. Mean values of ARA reported in sow colostrum or milk are 0.8 % and 0.6% of total fatty acids, respectively [24]. The effect of sow colostrum or milk on ARA metabolism need to further study. We previously discussed that exogenous addition of ARA can produce metabolites by cultured some bacteria or fungi in vitro [19]. However, whether the intestinal microbes could metabolize ARA to generate oxylipins in vivo remains to be further studied. Future work will be required to study the role of intestinal microbiota in ARA metabolism.

The intestine of the piglets is immature during the suckling period. At this stage, intestinal microbiota starts to be colonized, and the lymphoid cells begin to develop, potentially impacting the host metabolism [25,26]. It has been reported that ARA plays an important role in infant development and normal health. Free ARA modulates the function of ion channels, several receptors, and the immune system. Particularly, ARA has an essentiality in neonatal life and during development [7,27]. A growing number of studies have confirmed that ARA has been added to infant formulas and follow-on formulas to meet metabolic demand, and these studies have focused primarily on human. However, there are few studies on the physiological concentration of ARA and its oxylipins in the early life of piglets. While the majority of n-6 PUFA have proinflammatory properties as precursors of eicosanoids, some metabolites of ARA are proven to be involved in the regulators of innate lymphoid cells development and resolution of inflammation [8,28]. We reported here a lipidomics study of feces ARA-derived oxylipins in suckling piglets, and provided new information that could describe oxylipins changes of piglets over the suckling period. 

## 4. Materials and Methods 

### 4.1. Chemicals and Reagent

All oxylipins and deuterated internal standards were purchased from Cayman Chemical, Ann Arbor, MI, USA (Supplementary Table S1). HPLC-grade acetonitrile, isopropyl alcohol, acetic acid and methanol were purchased from Merck (Darmstadt, Germany).

### 4.2. Animals and Sampling

A total of 24 good health multiparous Landrace sows with an average parity of 4.74 were selected to our study. Sows were screened by the same inclusion criteria as those in Cheng et al. [29]. Sows had never received antibiotics before the study and kept in the same environment. Piglets were followed from farrowing until before weaning (about 21 days), and all piglets were housed with their respective sow in farrowing cages located in the same room with automated control of temperature. All sows were fed with the same lactation diet, which was formulated to meet or exceed the National Research Council [30] nutrient requirements for lactating sows. The piglets had no access to probiotics and antibiotics, importantly, no diarrhea and both sows and piglets had free access to water.

Fresh fecal samples were individually collected at 3 and 21 days after birth. Fecal samples collected directly from the rectum using sterile 15 mL centrifuge tubes from the piglets and then stored at −80 °C until being examined.

### 4.3. Analytical Condition of LC-MS/MS

Fresh fecal (50 mg weight) was frozen in liquid nitrogen, ground into powder. It was extracted with 1.0 mL cold methanol and was vortexed continuously for 5 min. The mixtures were kept in a 4 °C refrigerator overnight to ensure complete extraction. We then added 10 uL of mixed internal standard (1 μM). After the centrifugation using 5000 r/min for 10 min at 4 °C, the supernatant was collected and then evaporated to dryness under nitrogen gas stream, reconstituted in 100 μL of methanol: water (1:1, *v*/*v*). The solution was centrifuged and the supernatant was collected for LC–MS/MS analysis.

The sample extracts were analyzed in Metware (Wuhan, China) by using an LC-ESI-MS/MS system (HPLC, Shim-pack UFLC SHIMADZU CBM30A system; MS, Applied Biosystems 6500 Q TRAP system, MS, Foster City, CA, USA). 

The analytical conditions were as follows: HPLC: Waters ACQUITY UPLC HSS T3 C18 (2.1 mm × 100 mm, 1.8 μm, Foster City, CA, USA); mobile phase A, water with acetonitrile and acetic acid(40/60/0.002, *v*/*v*/*v*); mobile phase B, isopropyl alcohol: acetonitrile (50/50, *v*/*v*); gradient program, mobile phase A: mobile phase B, 99.9:0.1 V/V at 0 min, 45:55 V/V at 4.0 min, 1:99 V/V at 5.0 min, 1:99 V/V at 6.8 min, 99.9:0.1 V/V at 7.0 min; flow rate, 0.40 mL/min; temperature, 40 °C; injection volume: 10 μL. 

The ESI-MS/MS conditions were as follows: ion source, turbo spray; source temperature 550 °C; ion source gas I (GSI), gas II (GSII), curtain gas (CUR) set at 40, 40, and 35 psi, respectively, the collision gas was set to 5 psi. Triple quadrupole (QQQ) and LIT scans were acquired on a triple quadrupole-linear ion trap mass spectrometer (Q TRAP), API 6500 Q TRAP LC/MS/MS System, supplied with an ESI Turbo Ion-Spray interface, running in a positive ion mode and controlled by Analyst 1.6.1 software (AB Sciex, Foster City, CA, USA). 

The scheduled multiple reaction monitoring (MRM) was used in this study, which is based on these parameters including precursor ions (Q1), characteristic fragment ions (Q3), retention time, declustering potential (DP), and collision energy (CE) [31]. Briefly, the Q1 of target compounds were first searched by the quadrupole while any ions derived from diverse molecular weight compounds were screened. Many fragment ions were generated after an ionization collision chamber ionized and fragmented the precursor ions, which were then filtered by QQQ, and the desired characteristics of single-fragment ions (Q3) were selected. A specific set of MRM transitions was monitored for each period based on the metabolites eluted within this period [32,33]. The mass spectrum peak areas of all oxylipins were integrated after the metabolite mass spectrometry data of different samples were obtained, and the same metabolite mass spectrum peaks within the different samples were integrated for correction [34]. Finally, the recovery and accuracy data of LC-MS/MS analytical method were recorded (Supplementary Table S2). 

### 4.4. Identification of Differential Oxylipins

The metabolites were quantified by using the MRM model of the triple four-stage rod mass spectrometry [35,36]. All metabolites identify was carried out using Metware’s own and public metabolite database. An OPLS-DA model was established using multiple supervision methods. Differential accumulation of metabolites (DAMs) between samples was identified using orthogonal partial least squares discriminant analysis. Oxylipins with Log2|fold change| ≥ 1.0 and variable importance in projection (VIP) values ≥ 1.0 were considered as DAMs.

### 4.5. Chiral Analysis of 12-HETEs

Chiral LC-MS/MS separation analysis based upon the method of Neilson et al. [31]. Briefly, the chiral separation of 12(S) and 12(R)-HETE separation was performed by using a Chiral-Pak AD-RH analytical column (5 µm particle size, 2.1 × 150 mm) (Chiral Technologies, West Chester, PA, USA). LC-MS/MS conditions were as follows: The column was maintained at 40 °C, phase A: 95% H_2_O, 5% acetonitrile (ACN), 0.025% formic acid; phase B: 5% H_2_O, 95% ACN, 0.025% formic acid. The program was used with a flow rate of 0.2 mL/min. The linear gradient program was as follows: 50% B (0–10 min), 60% B (25 min), 100% B (27–30 min), and 50% B (31–40 min). Samples were maintained at 10 °C prior to injection and injection volume was 1 μL. Both *S* and *R* enantiomers of 12-HETE were calculated relative to the corresponding 12S-HETE-d8.

### 4.6. Statistical Analysis

Quantitative data are presented as mean values ± SD. Statistical analysis was performed using Tukey tests, and it was considered statistically significantly different at the *p* < 0.05 level.

## 5. Conclusions 

Our findings suggested that the level of oxylipins in feces was different between 3d and 21d after birth during the suckling period. These oxylipins were primarily derived from ARA, and ARA derived oxylipins generally present at the higher levels in the feces at early stage of sucking piglets. Particularly, 12-HETE was the highest concentration of feces. Considering that 12-HETE contained *S* and *R* enantiomers, we further detected the level of 12(S)- and 12(R)-HETE in feces by chiral analysis. There was no significant difference in 12(S)-HETE and 12(R)-HETE concentration at the same developmental stage of sucking piglets.

## Figures and Tables

**Table 1:** Oxylipins detected in feces of suckling piglets from 3d to 21d after birth.

Class	Number of Compounds
Arachidonic acid	21
Eicosapentaenoic acid	7
Docosahexaenoic acid	7
Linoleic acid	6
α-Linolenic acid	3
Dihomo-γ-Linolenic acid	2
γ-Linolenic acid	1
Mead acid	1

**Table 2:** Differential oxylipins in feces at 3d and 21d postnatally.

Oxylipins	Class	Fold Change	Log2FC	VIP
(±)5-HETE	ARA	0.16747	−2.57804	1.12796
(±)15-HETE	ARA	0.10813	−3.2092	1.12241
(±)12-HETE	ARA	0.05080	−4.29915	1.12629
11(S)-HETE	ARA	0.07390	−3.75832	1.19574
(±)9-HETE	ARA	0.09101	−3.45781	1.14467
11,12-EET	ARA	0.43841	−1.18963	1.09487
8,9-EET	ARA	0.38334	−1.38329	1.13316
LTB4	ARA	0.04309	−4.53647	1.12884
5-oxoETE	ARA	0.36316	−1.46131	1.09839
15-oxoETE	ARA	0.12029	−3.05542	1.17088
5,6-DiHETrE	ARA	0.40526	−1.30309	1.07919
DHA	DHA	0.34762	−1.52444	1.07157
(±)4-HDHA	DHA	0.13570	−2.88148	1.33369
(±)7-HDHA	DHA	0.05934	−4.07489	1.38459
14(S)-HDHA	DHA	0.02108	−5.56816	1.39682
(±)17-HDHA	DHA	0.01129	−6.46862	1.46415
PDX	DHA	0.23765	−2.07306	1.45008
16(17)-EpDPE	DHA	0.17971	−2.47626	1.14812
(±)5-HEPE	EPA	0.24632	−2.02137	1.05805
PGF3α	EPA	2.70297	−1.43454	1.17604
13-oxoODE	LA	0.16795	−2.57390	1.10934

**Table 3:** Chiral LC-MS/MS analysis of 12(S) and 12(R)-HETE enantiomers during suckling period.

Compound	Retention Time (Min)	[M-H]^−^ (*m*/*z*)
12-(S)-HETE	2.40	319.3 > 179.2
12-(S)-HETE-d8	2.39	327.3 > 184.3
12-(R)-HETE	1.87	319.3 > 179.2

## Data Availability

The data presented in this study are available in Supplementary Materials.

## Supplementary Materials

The following supporting information can be downloaded at: https://www.mdpi.com/article/10.3390/metabo12080702/s1.
